# Hollow Cone Electron Imaging for Single Particle 3D Reconstruction of Proteins

**DOI:** 10.1038/srep27701

**Published:** 2016-06-13

**Authors:** Chun-Ying Tsai, Yuan-Chih Chang, Ivan Lobato, Dirk Van Dyck, Fu-Rong Chen

**Affiliations:** 1 Department of Engineering and System Science, Tsing-Hua University, HsinChu 300, Taiwan; 2 Institute of Cellular and Organismic Biology, Academia Sinica, Taipei 115, Taiwan; 3 Department of Physics/ EMAT, University of Antwerp, Groenenborgerloaan 171, B2020 Antwerp, Belgium

## Abstract

The main bottlenecks for high-resolution biological imaging in electron microscopy are radiation sensitivity and low contrast. The phase contrast at low spatial frequencies can be enhanced by using a large defocus but this strongly reduces the resolution. Recently, phase plates have been developed to enhance the contrast at small defocus but electrical charging remains a problem. Single particle cryo-electron microscopy is mostly used to minimize the radiation damage and to enhance the resolution of the 3D reconstructions but it requires averaging images of a massive number of individual particles. Here we present a new route to achieve the same goals by hollow cone dark field imaging using thermal diffuse scattered electrons giving about a 4 times contrast increase as compared to bright field imaging. We demonstrate the 3D reconstruction of a stained GroEL particle can yield about 13.5 Å resolution but using a strongly reduced number of images.

The intrinsic barrier for biological imaging in electron microscopy is the fact that biological samples contain light element atoms that make them radiation sensitive and generate low phase contrast (signal/ noise). Furthermore, the electron microscope does not transfer phase contrast at low spatial frequencies. Several methods have been proposed to increase the contrast. Staining or labeling with heavy atoms methods can only increase the amplitude contrast by about 20%^1^. Phase contrast can be increased by using a large defocus, but this also causes blurring of the image due to defocus which strongly deteriorates the resolution. Another way to enhance the contrast is by using a Zernike phase plate (ZPP)^2^,^3^,^4^,^5^,^6^,^7^ or Boersch phase plate^8^,^9^,^10^ placed at the back focal plane of the objective lens which shifts the phase of the electrons over 90°. In this way the contrast transfer function of a microscope in the low spatial frequency range is altered from a phase contrast regime (sine-type of lens transfer function) to an amplitude contrast regime (cosine-type of lens transfer function), which allows to improve contrast at small defocus^7^. Hitherto, in the field of structural biology, single particle cryo-transmission electron microscopy (Cryo-TEM) has been the method of preference to reconstruct the three dimensional structure of biological macromolecules that cannot be obtained in crystalline form X-ray crystallography. This technique, however, relies on averaging the images of numerous particles from many different orientations to improve the poor signal-to-noise ratio and the low resolution. Although there are a few of demonstration cases with thin film type of Zernike^11^,^12^,^13^,^14^ and Volta-potential phase plate^15^,^16^,^17^ for contrast enhancement, their main drawback are unavoidable charging due to interaction of electron beam with carbon film phase plate that gives rise to image artifacts^3^ and non-controllable phase shift for image quantification^18^,^19^. A new imaging scheme that could to enhance the contrast without deteriorating the resolution and without artifacts by avoiding the extra electron scattering from interaction of electron beam/ phase plate film (device) would be very welcome.

It is known that high angle annual dark field imaging (HAADF) with high angle thermal diffuse scattered (TDS) electrons gives higher contrast than regular bright field imaging modes^20^,^21^. However, in HAADF STEM the electron beam is highly focused which obviously promotes damage to the sample. Hollow cone dark field (HCDF) imaging (Method, M-1) is in fact the reciprocal analogue of HAADF in which case the object is illuminated with a plane wave that is tilted over a fixed angle ***ϕ*** with respect to the optical axis and continuously precessed around a cone in such a way that the thermal diffuse scattering (TDS) that is maximal at high angles is collected within the objective aperture centered around the optical axis^22^,^23^. It is worth mentioning that HCDF is proposed as one of the most feasible technique for outrunning radiation damage using the snapshot electron beam source^24^. Figure 1 shows the principle of reciprocity in optics for HAADF (Fig. 1a) and HCDF (Fig. 1b). As shown in Fig. 1b, by tilting the angle ***ϕ*** of the incident beam with respect to the optical axis one can choose the angular range of the electron beams that will contribute to the image. And by precessing the beam around the optical axis and integrating the incoherently TDS scattered signals, the symmetry is restored and the incoherent image of each atom is then independently added to the final image. This result simplifies the interpretation of the high resolution images and it is very suitable for tomographic reconstruction algorithms. The objective aperture also eliminates the tilted incident beam so as to select mainly the TDS electrons for the formation of the image. Furthermore, it is possible to adapt any electron microscope to such imaging mode without special customization. Another advantage of this incoherent imaging mode is that only the TDS electrons are selected so that no further electron scattering is introduced into the beam path, which avoids possible charging problems and additional phase shift (elastic scattering) that of the film type of phase plate^3^,^18^,^19^. The high-resolution information is minimally affected by the lens aberrations and is transferred in the amplitude contrast image at its full signal strength.

In this paper, we report the potential power to use HCDF with TDS electrons as a new high contrast imaging mode for 3D biologic imaging of single particle reconstruction using a strongly reduced number of images. The proof of the concept will be demonstrated experimentally with negative stained chaperonin protein GroEL which is a standard protein with a well-known structure^25^,^26^. By using sets of electronically controlled scanning coils (Method, M-1) the incident beam can be scanned conically and the objective aperture is centered around the optical axis of the EM in HCDF mode in two different TEMs (FEI Tecnai F20 and JEOL JEM-2010F).

## Results

### TDS and optimum imaging conditions for HCDF

As the high energy electrons interact with the atoms, in reality, the atoms of the object are not still but they thermally oscillate around an average position during the recording of the image. It is generally believed that this reduces the large angle scattering and thus also the high spatial frequencies so as to have only a minor influence on the image contrast. It is pointed out that scattering of electrons by thermally vibrating atoms is much stronger than intuitively expected because a fast electron sees a still atom displaced from its equilibrium position and interacts with the strong electrostatic potential of the nucleus of the atom which predominantly scatters at high angles^27^. Let us for simplicity consider the case of one particular atom with projected electrostatic potential 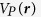
and with ***r*** is taken in the plane of projection. In Fourier space the scattering factor of the still atom 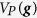 is given by the Mott-Bethe formula


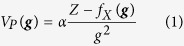


where 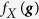 is the X-ray scattering factor and Z is the atomic number, and α = 2me/ħ^2^.

Note that the “tail” of this factor decreases only slowly with increasing ***g***, which is a consequence of the singularity of the electrostatic potential of the nucleus of the atom. Since the atom vibrates around its equilibrium position we can define a kind of averaged potential 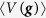. In Fourier space the scattering factor of the atom 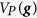, is then multiplied by a Gaussian “Debye-Waller” factor which is well known in X-ray crystallography.


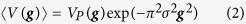


***σ***^2^ is the mean square displacement of the atom. The error that is usually made in electron diffraction theory is that the electron scattering factors of the atom are also systematically multiplied by Debye-Waller factors as if the atoms are replaced by their thermal average. However this approximation which stems from X-ray diffraction is not valid for electron diffraction. Indeed every electron sees a still atom (frozen atom) and the averaging over the different atom positions has to be done at the level of the detection of the images or diffraction patterns^28^. Since the atom has a very sharp potential in its center the electron will scatter appreciably at high angles. This high angle scattering does not disappear on averaging. If on the other hand the atom is “blurred” by a Debye Waller factor, the high angle scattering is artificially reduced so that the thermal diffuse scattering (TDS) is underestimated. The TDS is generated by the difference between 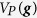 and 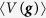.


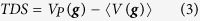


TDS scattering has a maximum in Fourier space at a spatial frequency that is the inverse of the mean square displacement of the atom motion. The total incoherent (TDS) intensity is roughly given by 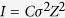 which is not only proportional to *Z*^2^ (*Z* is the atomic number) but also to the mean square displacement ***σ***^2^ of the atom^27^ which is inversely proportional to the 1/M^29^,^30^ (or 1/Z). Hence TDS signal gives Z-dependent imaging that can be important especially for light atoms in soft matter where the interatomic bonds are generally loose and where the mean square displacement can be much larger than in inorganic perfect crystals. TDS scattering can even be stronger than the elastic signal at high angles. The TDS signal of a carbon atom is maximal around at smaller spatial frequency of the object of 0.5 Å^−1 ^27. Furthermore, the TDS signal is linear in the mass-thickness and easy to interpret and hence very suited for tomography. Figure 2 shows a plot of the angular distribution of the elastic scattering (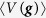, coherent contribution) of an averaged carbon atom, and its TDS (incoherent contribution) for various values of ***σ***. For very low ***σ*** (0.1 Å), which is typical for inorganic crystals, the TDS contribution is relatively small and distributed over a very large area in Fourier space. However, for very large ***σ*** (0.3 Å) as typical for soft matter, the TDS contribution is comparable to the coherent contribution and peaked at relatively low spatial frequencies (~0.64 Å^−1^).

For a weak phase object 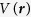, the electron wave-function, after transmission through the objective lens the image wave 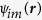 thus becomes


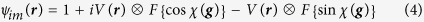


where *χ*(***g***) is the phase shift of the objective lens,


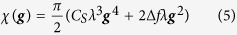


including the spherical aberration (*C*_*S*_), wave length (*λ*) and defocus (*Δf*)^31^. Here we ignore the damping envelope function and the aperture function of the objective lens. Now we neglected the second order term and for bright field image intensity


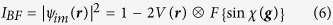


While for the HCDF, the image intensity can be written as:


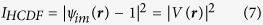


As it is implicitly shown from the equations (6 and 7), the contrasts of bright field and the HCDF are given to be 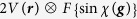 and 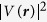. For the beams that are collected by the aperture the angle with the optical axis is relatively small so that 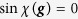 and 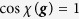 for all relevant spatial frequencies ***g***. The contrast of the HCDF is obviously higher than that of the bright field image. In case the electron microscope is equipped with a spherical aberration corrector and is operated close to zero focus, this range of spatial frequencies (and thus also the radius of the aperture) can be extended to higher resolution. Equation (7) shows that even with a non-*Cs* corrected EM, one can make the transfer function sufficiently constant over the objective aperture so as to generate amplitude images with atomic resolution. For a microscope with a 20 μm objective aperture (equivalent to 0.3 Å^−1^) and *C*_*S*_ = 2 mm, the optimum focus for HCDF (Δ*f*) is given by *C*_*S*_*λ*^2^*g*^2^/*2* = *−*57 nm.

### Analysis of experimental images and single particle reconstruction

The contrast of the HCDF depends on the tilt angle ***ϕ*** and the aperture size β^23^. From Fig. 2, the maximum of the elastic signal 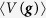 and the TDS signal are in a different angular range. It can be seen that for soft matter (***σ***~0.3 Å), the total intensity is dominated by the TDS incoherent contribution which has the maximum TDS scattering around a spatial frequency of g~0.64 Å^−1^ which is equivalent to an optimum tilting angle ***ϕ*** of about 16.12mrad. The spatial frequency of TDS is extended between 0.4 Å^−1^ and 0.8 Å^−1^ so that one can use an objective aperture with a radius of about 0.25 Å^−1^ which is equivalent to a collection angle β of 6.3mrad. The focal length f of the microscopes are 2.3 mm for JEOL and 2.7mm for FEI. The optimum size of the aperture β is therefore calculated to be around 20 μm~40 μm using tangent equation, 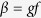. The precession frequency of electron beam in our experiment is set to be about 58.1Hz (Method, M-1). Since the precession rate of the electron beam is around 60Hz, the dose can be controlled to be the same as that used in the bright field imaging mode for cryo-EM. It is worth mentioning that the total dose on the sample depends on the dose rate (intensity of incident beam, electrons/Å^2^sec) and the exposure time (second). In our experiment, the total doses are the same for bright field (phase contrast) and HCDF, since the images of both imaging modes are recorded with the same intensity of electron beam and same exposure time. In our experiment, the total dose has nothing to do with the aperture since the same size of aperture is used for both imaging modes. The typical dose rate and exposure time we used is about 20–30 electrons/Å^2^sec and 1 second. Therefore the total dose is around 20–30 electrons/Å^2^ in our experiment which falls nicely into requirement for the single particle reconstruction^25^. The total dose in our HCDF image was set at 20–30 electrons/Å^2^, which is 4–6 times lower than total dose of cryo-STEM tilt series tomography^20^,120–150 electrons/Å^2^, although the dose of each STEM tilt series image was set at 6 electrons/Å^2^. Furthermore, the dose efficiency is relatively inefficient per incident electrons in HAADF^32^ and HCDF, the measured dose efficiency in our case is about 4.56% which is consistent with simulation result of HAADF^32^.

It is therefore expected that the HCDF imaging mode proposed here can be equally applied for cryo-EM for 3D single particle reconstruction. The GroEL is an 800kDa molecular chaperonin which comprises two rings of seven subunits^33^,^34^. The structure had been published by Wah Chu at 4.0 Å resolution^25^,^26^. Therefore, it is suitable as a standard benchmark molecular for 3D single particle reconstruction via HCDF and image simulation. The GroEL was negatively stained with uranyl acetate. Figure 3a–d shows the bright field and HCDF images of the GroEL particles (Method, M-2), recorded by a FEI Tecnai F20 with ***ϕ*** = 17mrad, with aperture radius 20 μm which is equivalent to a collecting spatial frequency range between 0.38Å^−1^ and 0.97 Å^−1^. Figure 3e–h shows the results recorded by a JEOL JEM-2010F with ***ϕ*** = 16.12mrad, aperture radius 10 μm corresponding to a collecting spatial frequency range between 0.47 Å^−1^ and 0.82 Å^−1^. Note that the contrast of the bright field image is inverted to allow a quantitative comparison with that of HCDF images. The Rose contrast criterion *C*_*R*_ can be quantified as^4^:


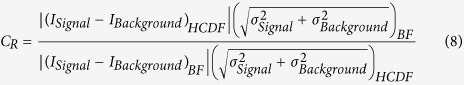


where *I*_*Signal*_ and *I*_*Background*_ are the averaged intensity from the regimes of GroEL and the carbon supporting film, respectively, while the *σ*_*Signal*_ and the *σ*_*Background*_ are the standard deviation of this two parts, respectively. The intensity profiles (Fig. 3i,j) across GroEL and background are averaged from a corresponding area of 200×10 pixels^2^ in the images (Fig. 3c,d,g and h). The contrast improvement is visually evident as shown in line profiles (Fig. 3i,j). The contrast of the HCDF image is, respectively, about 4.30 and 4.40 times higher than the corresponding bright field images for these two cases.

It is therefore expected that this significant improvement in contrast will reduce the number of particles that is needed for the 3D reconstruction. A number of particles have been selected and single particle analysis has been performed straightforwardly with the EMAN2^35^,^36^ software suit. The refined structure was obtained based on approximately 8800 particles, picked directly from 200 raw HCDF images as shown in Fig. 3. Figure 4a,b show the relationship between the classes average and the projections from the initial model provided by EMAN2. Figure 4c,d show the EM density maps refined by the result of classes average and the initial model by using Chimera^37^. The EM density shows the seven-fold symmetry on top view and the seven-fold axis in the vertical direction. Four different density areas are found on the side view, which line up with the inner and outer subunit domains to give the four continuous layers. Figure 4e shows the EM density map setting with an isocontour threshold to include the total mass of the GroEL molecular complex. Furthermore, we calculated the resolution of the refined structure at ~13.6 Å by Fourier shell correlation^38^ using the 0.5 criterion in the EMAN2 software suit (Fig. 4e). As shown in Fig. 4f, this is far better than the resolution of about 20 Å that has been obtained thus far in the literature for negatively stained 3D GroEL^39^,^40^,^41^.

## Discussion

We have experimentally demonstrated that thermal diffuse scattering TDS electron can be used in structural biology in HCDF mode with optimum tilting angle and objective aperture, and this experimental finding is confirmed by the theoretical simulation with a model of a GroEL protein on a 9 nm thick carbon film. As shown in Fig. 5a, totally there are 50211 atoms in the model including the amorphous carbon film and 1274 uranium atoms stained GroEL. The model of GroEL itself contains 7364 atoms which can be downloaded from the Protein Data Bank under number PDB ID: 3C9V^25^,^42^. In this paper, tilt angle and objective aperture are used to control the collection scattering angle at the range of 11.8~20.48mrad which provides the signal of HCDF comes mainly from carbon atoms. Simulated bright field (left) and HCDF (right) images of GroEL with atomic displacement 0.3 Å and with a tilt angles of 16.12mrad and a collecting angle between 11.8~20.48mrad are displayed in Fig. 5b. Figure 5c shows the class average version of the experimental images recorded in bright field and HCDF modes by EMAN2 software .To reveal the subtle difference in contrast, all images are displayed in pseudocolor mode. The background intensity *I*_*Background*_, is subtracted from the simulated bright-field image and the HCDF are the images are then normalized with the square root of the standard deviation. Figure 5d shows the intensity profile across the simulated GroEL intensity along the dashed line in Fig. 5b. As shown in the Fig. 5d, the contrast of the simulated HCDF image is about 7.2 times higher than that of the bright field image, according to the equation (8). The well-known contrast mismatch between image calculations and experiments can be related to the “Stobbs factor” which can be explained by linking the thermal diffuse scattering TDS to electron beam-induced object excitation and relaxation processes^43^. The higher contrast ratio deduced from the simulated images gives a Stobbs’s factor which in our case is around 1.8 (=7.2/4). This ratio may depend on the dose rate due to “potential softening^44^. Although, another possible reason for the contrast mismatch may be that the thickness of the carbon supporting film is much thicker than in our simulation model, our results here could provide empirical evidence to prove that the contrast enhancement in HCDF mode can be used effectively for single particle 3D reconstruction with a strong reduction in the required number of particles.

As discussed above, the dose of HCDF imaging mode can be controlled to be the same as that of bright field by controlling the beam intensity and exposure time. In bright field mode (phase contrast), however, since the higher intensity of the transmission beam is used in bright field, and in HCDF mode the thermal diffuse scattering (TDS) is collected, the bright field image gives slightly better signal/noise (S/N) as compared with that of HCDF. However, the higher contrast (signal/ background) in HCDF image can compensate the fact of lower S/N that allow to reconstruct 3D single particle tomogram with even reduced number of image. It is therefore expected that the HCDF imaging mode proposed here can be equally be applied in cryo-EM for 3D single particle reconstruction.

## Conclusion

We have shown that it is possible to obtain high resolution images using TDS electrons. The image contrast is amplitude contrast. The contrast can be about 4 times higher than that of a corresponding bright field image, even for soft matter. When the imaging is done with a flat transfer function, using a small defocus and a *Cs* corrected electron microscope and an appropriate aperture, the resolution can approach the limits of the electron microscope. Furthermore every atom contributes its own image intensity independently which makes the images easy to interpret and suitable for tomographic schemes.

Furthermore, since the total dose of the HCDF imaging mode can be maintained as low as that of bright field, we believe that the same HCDF imaging mode can be applied to cryo-EM for 3D protein reconstruction via a single particle scheme.

## Method

### M-1: The Set-up of Hollow Cone Dark Field (HCDF) Imaging

There are several methods to produce hollow cone illumination (HCI) in an electron microscope. Riecke^45^ and Dupouy^46^ proposed to use an annular condenser aperture. Each element of the cone of illumination is inclined to the optical axis. For dark field imaging, the diameter of the objective aperture is chosen to be just small enough to block the hollow cone primary beam which results in contrast produced by scattered electrons. Hollow cone imaging with an annular aperture produces an image which is an incoherent superposition of separated scattered beams. The hollow cone illumination is axially symmetric, hence it is possible to correct for the objective lens astigmatism since a standard objective lens aperture is employed. However, one of the main drawback for using an annular condenser aperture is that the tilt angle and the annulus are fixed and cannot be adjusted.

**Figure 1:**
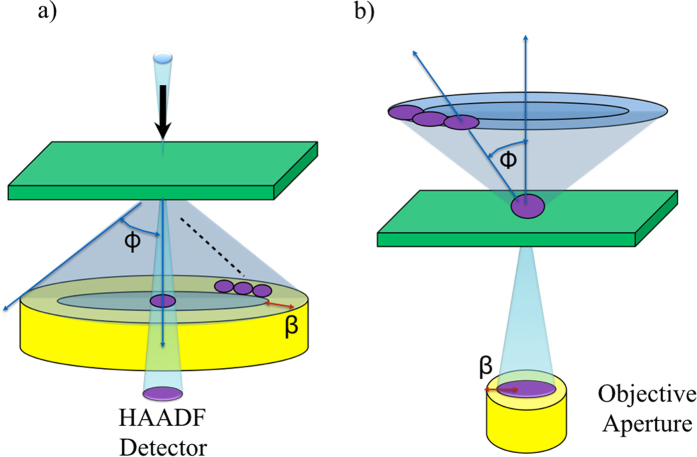
The reciprocity between high angle annual dark field (HAADF) and hollow cone dark field (HCDF). (**a)**, HAADF, the diffraction angle is *ϕ* such that the maximum TDS can be collected with annual detector (**b)**, HCDF, the tilt angle *ϕ* is chosen such that the diffraction at high angle *ϕ* containing the maximal TDS signal that can be collected within the objective aperture centered around the optical axis. *β* is the collecting angle which defines the size of detector.

**Figure 2:**
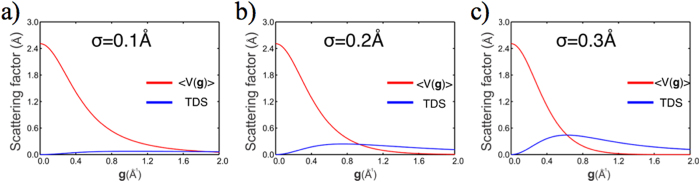
Frozen atom image calculation for a vibrating carbon atom. Elastic scattering (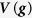, red curve) and TDS (blue curve) scattering factors for different displacements: (**a**), *σ* = 0.1Å, (**b**), *σ* = 0.2 Å and (**c**), *σ* = 0.3 Å.

**Figure 3:**
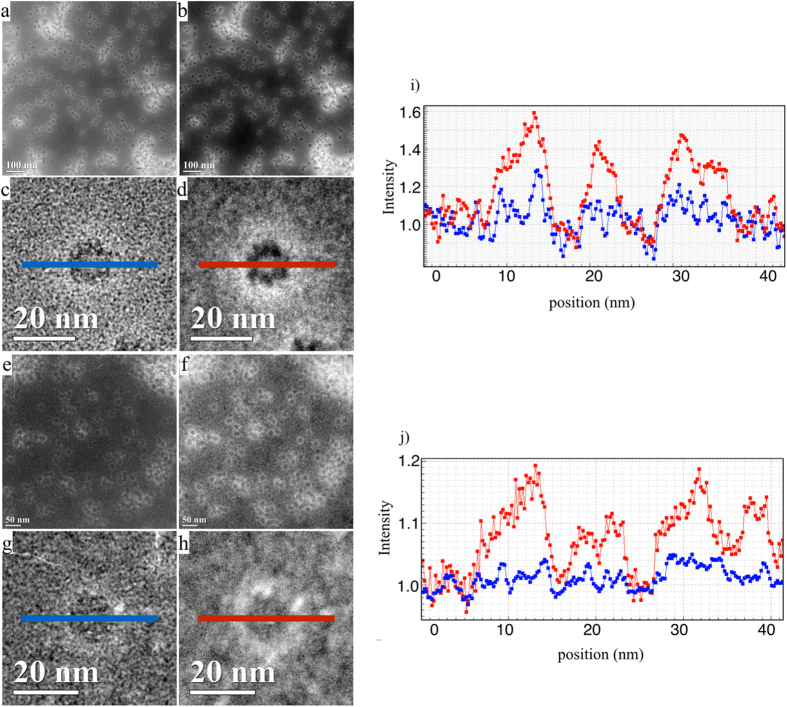
Quantitative comparison of bright field and HCDF. (**a**,**b)** show the bright field and HCDF images of GroEL, which were recorded by a FEI Tecnai F20 with a beam tilt angle of *ϕ* = 17mrad. A 20 μm radius aperture was used which corresponds to a collecting TDS signal between 9.58~24.40mrad (0.38~0.97 Å^−1^). (**c**,**d)** selected GroEL particles form both image modes. (**e**,**f)** show the bright field and HCDF images, recorded using a JEOL JEM-2010F with beam tilt angle *ϕ* = 16.14mrad. A 10 μm radius aperture was used which correspond to a collected TDS signal between 11.8~20.48mrad (0.47~0.82 Å^−1^). (**g**,**h)** selective GroEL particle form both image modes. (**i**,**j)** are the intensity profiles across the particles in (**c**,**d**,**g**,**h**), respectively.

**Figure 4:**
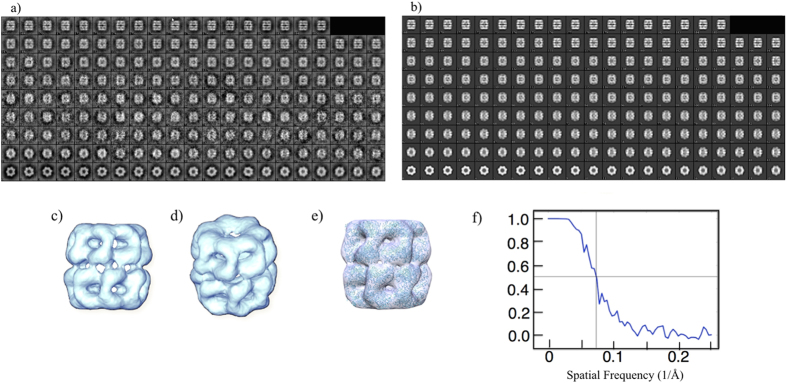
Reconstructed EM density maps of GroEL from HCDF images. (**a**), the result of classes average form the 8800 selective GroEL particles in 200 raw HCDF images (**b**), the projections from the initial model generated by EMAN2. (**c**), side view of EM density map (**d**), inclined view of the EM density map (**e**), shows the fitting between the EM density map and the molecular complex of GroEL downloaded from the Protein Data Bank PDB ID: 3C9V (**f**), the Fourier shell correlation curve to show the 13.57 Å resolution of the constructed EM map using the 0.5 criterion.

To overcome this drawback, we form a hollow cone image as shown in Fig. 1b which is temporally incoherent with applying voltage modulation to both x and y tilt coils simultaneously with a standard condenser aperture. In other words, a dark field image is composed of spatial incoherent images where each image is recorded simultaneously during the time required to produce the micrograph. As shown in Fig. 6, the electronic devices are installed in the position near a deflector coil of the second condenser. The cone azimuth of HCI can be adjusted with voltage modulation.

### M-2: Simulation of the HCDF image for the GroEL protein

**Figure 5:**
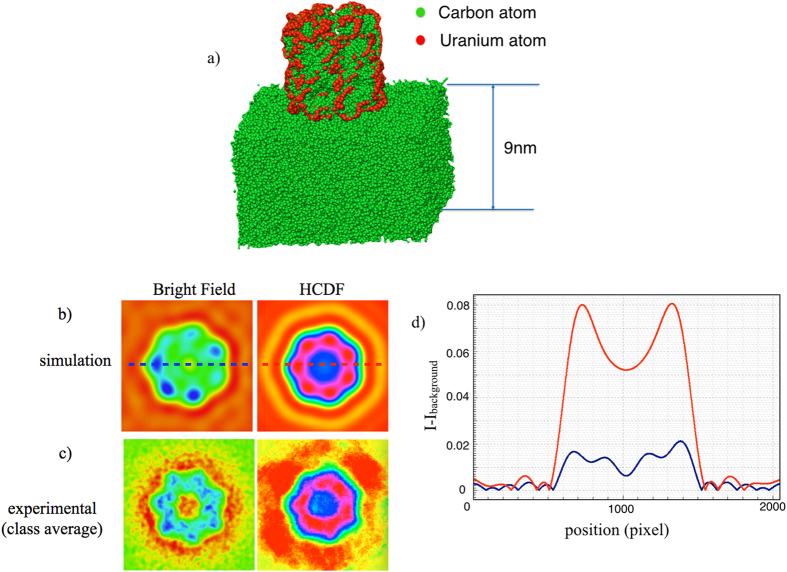
Quantitative contrast analysis of bright field and HCDF simulated images. (**a**), structural model of a GroEL protein on a 9 nm thick carbon film. The red atoms in the model are the Uranium atoms and the green atoms are carbon atoms. The cylindrical object is the GroEL protein. The thickness of the supporting carbon film is 9 nm. (**b**), the simulated images of bright field (left) and HCDF (right). (**c**), the class averaged experimental images of bright field (left) and HCDF (right). (**d**), the intensity profiles across the simulated images along the dashed lines in the 5b.

**Figure 6:**
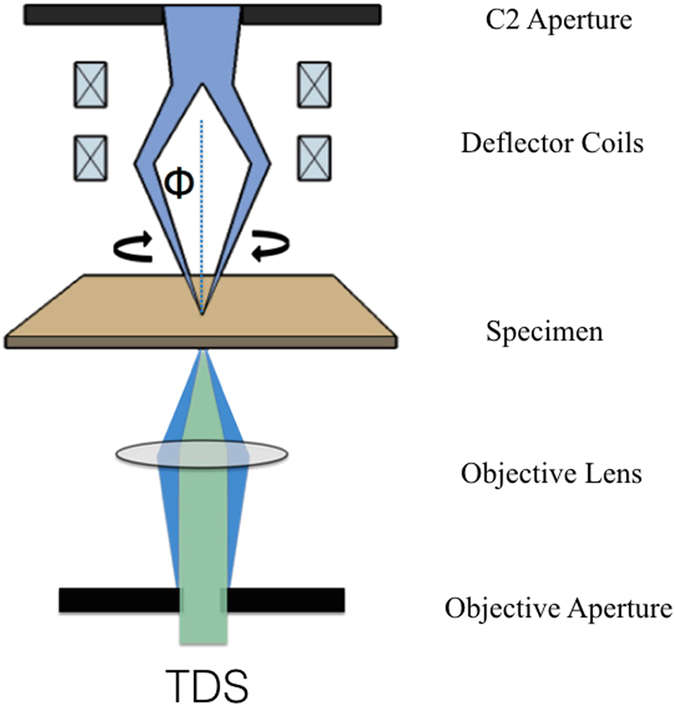
Electronic set-up of hollow cone imaging. Schematic drawing shows the deflector coils configuration for precessing the electron beam and the objective aperture to collect the TDS for hollow cone dark field.

The GroEL model was downloaded from the Protein Data Band (PDB) #3C9V and covered with 1274 uranium atoms (red atoms in Fig. 5a) on the surface and supported by an amorphous carbon thin film layer of 9 nm thickness. The structure model has unit cell parameters of a = b = 200 Å, c = 250 Å and α = β = γ = 90°. The thermal vibration of the atomic displacement ***σ*** is set to be 0.3 Å. Both bright field and HCDF modes image simulation is processed in MacTempasX suit^47^. The electron microscope parameters used are given as follows: acceleration voltage (**E**_**0**_ = 200KeV), spread of defocus (***Δ*** = 15 nm), semi-convergence angle (***α*** = 1.5mrad), spherical aberrantion (*C*_*S*_ = 1.0 mm), defocus (Δ*f* = −57 nm), objective aperture radius (***β*** = 0.17 Å^−1^) and tilt angle (***ϕ*** = 16.14mrad) for HCDF mode. These parameters correspond to a collected angle between 11.8~20.48mrad for collecting the TDS signal.

## Additional Information

**How to cite this article**: Tsai, C.-Y. *et al*. Hollow Cone Electron Imaging for Single Particle 3D Reconstruction of Proteins. *Sci. Rep*. **6**, 27701; doi: 10.1038/srep27701 (2016).
